# Inverse relationship of Rho kinase and myosin-light chain kinase expression in the aging human detrusor smooth muscle

**DOI:** 10.1186/s12894-015-0098-2

**Published:** 2015-10-15

**Authors:** Timo Kirschstein, Theresa Sahre, Karoline Kernig, Chris Protzel, Katrin Porath, Rüdiger Köhling, Oliver W. Hakenberg

**Affiliations:** Oscar Langendorff Institute of Physiology, University of Rostock, Gertrudenstrasse 9, 18057 Rostock, Germany; Department of Urology, University of Rostock, Rostock, Germany

**Keywords:** Aging, Detrusor, Human, Real-time RT-PCR, ROCK-to-MLCK ratio

## Abstract

**Background:**

Rho kinase (ROCK) and myosin-light chain kinase (MLCK) are key enzymes in smooth muscle contraction. Previous data have suggested that ROCK contribution to human detrusor contraction is increasing with age. Here, we have analyzed the transcriptional expression of Rho kinase isoforms (ROCK1 and ROCK2) as well as MLCK in the aging human detrusor smooth muscle obtained from resected tissue.

**Methods:**

Small pieces of macroscopically healthy human detrusor smooth muscle (urothelium-free) were prepared for quantitative real-time reverse transcriptase polymerase chain reaction (RT-PCR). Transcript expression (mRNA level) of the target genes ROCK1, ROCK2 and MLCK was normalized to three common reference genes (glyceraldehyde-3-phosphate dehydrogenase, β-actin, phosphoglycerate kinase 1).

**Results:**

We found that across all ages the expression level of ROCK (i.e. ROCK1 and ROCK2 together) was almost equal to that of MLCK in the human bladder. Further, ROCK2 showed a significantly higher expression level than ROCK1. Among all subjects, there was no significant correlation of any single target gene to age, but expression levels of ROCK and MLCK were inversely correlated. Moreover, the within-subject analysis revealed that the ROCK-to-MLCK ratio showed a significantly negative correlation to age. Thus, within a given subject, there is a relative ROCK down-regulation and concomitant MLCK up-regulation.

**Conclusions:**

Together with previous data in human detrusor specimens showing increased ROCK contribution to detrusor contraction, we speculate that the drop of the ROCK-to-MLCK ratio may occur as an attempt to compensate for the increased Rho kinase activity.

## Background

Age-related changes in urinary bladder contractility are believed to contribute to dysfunctional micturition such as overactive bladder syndrome (OAB). This is a common urological condition associated with urgency, with or without urge incontinence, and increased micturition frequency and nocturia [1]. OAB prevalence increases with age [2], and in order to find new therapeutic strategies for the management of OAB it is crucial to unravel the pathophysiological mechanisms leading to increased bladder contractility. Hence, it is obvious that there is a need for studies on contraction and relaxation mechanisms in the aging human urinary bladder. During the last decade several groups have focussed on the classical adrenergic and cholinergic mechanisms of detrusor motility. For instance, detrusor relaxation following sympathetic β-adrenoceptor activation was not altered during aging [3], the same was true for cholinergic responses [4]. Although there is no age-related correlation, current medication is still based on antimuscarinics and β-sympathomimetics. On the other hand, purinergic receptor-mediated contraction increased with age [4] and may thus be regarded as an attractive candidate mechanism. Beyond receptor activation, intracellular contraction mechanisms could also be accessible targets for pharmacological manipulation. We have recently addressed the key enzymes Rho kinase (ROCK) and myosin-light chain kinase (MLCK) in the aging human bladder and could demonstrate that ROCK contribution to detrusor contractility was positively correlated with age, while MLCK contribution was not altered during aging [5]. Therefore, we hypothesized that transcriptional up-regulation of Rho kinase could be responsible for this age-related effect. To this end, we performed quantitative real-time reverse transcriptase polymerase chain reaction (RT-PCR) in order to determine the mRNA expression levels of the two isoforms of Rho kinase (ROCK1 and ROCK2) and MLCK in detrusor smooth muscle tissue obtained from patients undergoing cystectomy.

## Methods

### Preparation of detrusor smooth muscle strips

Human detrusor samples were obtained from 41 patients with an average age of 67 ± 10 years (mean ± S.D.; range 46–84 years old; 33 male and 8 female patients, Table 1). These specimens (1–2 cm width) were prepared from surgically resected bladder wall obtained from patients who underwent cystectomy. The indication for cystectomy was bladder cancer (urothelial carcinoma) with or without prostate cancer in 35/41 cases, other malignancies with or without infiltration of the urinary bladder in 4/41 cases and neurogenic bladder in 2/41 cases (Table 1). Care was taken to dissect only macroscopically healthy bladder wall tissue for this study, but the anatomical origin could not be standardized due to requirements for routine histopathological examinations especially in radical cystectomy. The specimens always contained urothelium which was removed before small pieces were cut for the PCR analysis. Thus, the tissue used in this study was always obtained from within the detrusor smooth muscle. All in vitro experiments with human material performed in this study were approved by the local ethics committee (University of Rostock), and the informed consent to participate in this study was obtained from each patient.

**Table 1:** Patient data

No.	Age	Sex	Diagnosis and cystectomy indication	Type of surgery	History
1	68	m	Bladder cancer G2 pT2b pN0 (0/9) L0 F0 R0 cM0	Radical cystectomy/neobladder	S/P 1× TUR bladder G3 pT2a
2	66	w	Bladder cancer pT1 G3 pN0 L0 V0 R0 M0	Radical cystectomy/Mainz Pouch I	S/P 1× TUR bladder G2 pT1 and Cis
3	61	m	Bladder cancer pT3a pN0 cMx R0 G3 Carcinoma left renal pelvis pT2 pN3 cMx R1 G3 Prostate cancer pT1a pN0 cMx Gleason 2 + 3 = 5	Radical cystectomy and nephroureterectomy left/ileal conduit	S/P 11× TUR bladder G2 pTa and Cis, at least pT2 G3
4	80	m	Bladder cancer pT2b (is) pN0 cM0 R0 L0 V0 G3 Prostate cancer: pT2a pN0 (0/13) R0 L0 V0 Gleason: 3 + 3	Radical cystectomy/ileal conduit	S/P 1 × TUR bladder G3, pT2a
5	68	m	Bladder cancer pT1 (is) pN0 (0/12) G3 R0 L0 V0	Radical cystectomy/ileal conduit	S/P 1× TUR bladder pT1 G3 and pTa G2
6	62	m	Bladder cancer pT2b (is), pNo (0/20) G3 R0 L1 V0 cM0	Radical cystectomy/ileal conduit	S/P 1× TUR bladder pT2a G3
7	73	m	Bladder cancer (with adenoid vegetations) G3 pT1 pN0 (0/11) L0 V0 R0 M0 Prostate cancer G2 pT2c PN0 L0 V0 R0 M0 Gleason 3 + 2 = 5	Radical cystectomy/ileal conduit	S/P 1× TUR-prostate (no malignancy) S/P 1x TUR-bladder pT1 G3
8	46	m	Bladder cancer G2 pTa pN0 (0/18) L0 V0 R0 M0 and renal failure (on dialysis)	Radical cystectomy/ileal conduit	S/P 1× TUR-bladder (necrotic urothelial carcinoma)
9	84	w	Bladder cancer G4, pT4a pN0 (0/9) M1 (PER) L1 V0 R1 (with sarcomatoid differentiation)	Radical cystectomy with cutaneous ureterostomy	S/P 6× TUR-bladder rpTa-1 G1-2, at least G3 pT2
10	50	m	Prostate cancer 4 + 5 = 9; pT4 pN1 (8/22) L0 V0 R0 cM0 (with bladder infiltration)	Radical cystectomy/ileal conduit	S/P prostate biopsy pT1c Gleason 5 + 4 = 9 and suprapubic catheter after overflow bladder
11	60	m	Bladder cancer pT1 pN0 cM0 R0 G2 (high grade)	Radical cystectomy/ileal conduit	S/P 1× TUR bladder pTa G2 and pT1 G2
12	80	w	Bladder cancer pT1 pN0 cM0 R0 G2 (high grade)	Radical cystectomy with urethrectomy/ileal conduit	no history available
13	68	m	Neurogenic bladder (contracted bladder) Prostate cancer pT2c R0 L0 V0; Gleason 3 + 3 = 6	Radical cystectomy/ ileal conduit	Incomplete paraplegia Th4 Polyneuropathy due to alcoholism S/P suprapubic catheter
14	74	m	Bladder cancer pT1 pN2 (2/17) G3 R0 L1 V0	Radical cystectomy/ileal conduit	no history available
15	59	m	Bladder cancer pT3b pN2 (2/12) G3 R0 L1 V0	Radical cystectomy/neobladder	S/P 2× TUR-bladder pT1 G2-3 (adenocarcinoma)
16	60	m	Bladder cancer pTis pN0 (0/24) R0 M0 Renal pelvic cancer pT3 G2 R0 N0 L0 V0	Radical cystectomy/ileal conduit and left nephrectomy	S/P 1×TUR-bladder pTis with urothelial carcinoma G2-3 and urothelial carcinoma left renal pelvis G1 pTa
17	57	m	Bladder cancer G3 pT1 pN0 (0/26) L0 V0 R0 cM0	Radical cystectomy/neobladder	S/P 3× TUR-bladder G3, pTa and mrpTis
18	71	m	Bladder cancer G3 pT3b pN2 (9/20) L1 V1 R0 cM1	Radical cystectomy/ileal conduit	S/P 7× TUR-bladder pTa G2 and pT1G3 and rpT1G3
19	74	m	Bladder cancer G3 pT2a pN0 (0/5) L0 V0 R0 cM0 Prostate cancer pT2 pN0 M0 G2	Radical cystectomy/ ileal conduit	S/P 1× TUR-bladder mpT1 G3
20	72	m	Bladder cancer pTX pN2 (3/12) G3 R0 cM0	Radical cystectomy/ileal conduit	S/P TUR-bladder T2a G2-3
21	75	m	Bladder cancer G3 pT3b pN0 (0/14) L1 V0 R1 cM0 Prostate cancer Gleason: 4 + 5 = 9; G3 pT2c pN0 L0 V0	Radical cystectomy/ileal conduit	S/P 1× TUR-bladder T2 G3, renal failure
22	56	m	Bladder cancer G3 pT3a pN3 L1 V1 pM1 (LYM) Prostate cancer Gleason 2 + 3 = 5; G2 pT2c	Radical cystectomy/ileal conduit	S/P TUR-bladder T2 G3
23	77	m	Bladder cancer G3 pT1 pN0 (0/13) L0 V0 R0 cM0 Prostate cancer Gleason 3 + 4 = 7; G3 pT2a pN0 L0 V0 R0 M0	Radical cystectomy/ileal conduit	S/P 4× TUR-bladder rpT1G2 and pTa G2
24	84	m	Bladder cancer G3 pT2a pN0 (0/17) L0 V0 R0 cM0	Radical cystectomy/ileal conduit	S/P 3× TUR-bladder pTa G1-2 and at least pT2 G3
25	56	w	Bladder cancer pT1 (is) pN0 cM0 R0 G3 V0 L0	Radical cystectomy/Mainz Pouch I	S/P 1× TUR-bladder pT1 G3 and mpTis
26	80	w	Bladder cancer G3 pT1 (is) pN0 (0/21) Mx V0 L0 R0	Radical cystectomy/ileal conduit	S/P 1× TUR-bladder G3 pT1
27	55	w	Urethra cancer pT2 pN0 (0/22) G2 R0 L0 V0 cM0 (squamous cell carcinoma)	Radical cystectomy/Mainz Pouch I	S/P 1× TUR-urethra (squamous cell carcinoma pTis)
28	52	m	Bladder cancer pT3b pN2 (9/20) M1 (lymph) G3 R0 L1 V0 Prostate cancer pT2c pN0 cM0 R0 L0 V0 Gleason 3 + 4 = 7	Radical cystectomy/ileal conduit	S/P 1× TUR-bladder G3 pT2
29	56	m	Bladder cancer pT3a pN0 (0/25) G3 R0 L1 V0 cM0	Radical cystectomy/neobladder	S/P 1× TUR-bladder G3 pT2 (is)
30	56	m	Prostate cancer Gleason 4 (80 %) + 3 = 7; G3 pT4 pN1 (4/12) L1 V0 R1 cM0 (with bladder infiltration)	Radical cystectomy/ileal conduit	no history available
31	76	w	Bladder cancer pT3b pN1 (1/18) G3 R0 L1 V0 cM0	Radical cystectomy/ileal conduit	S/P 1× TUR-bladder pT2 G3 S/P TFS (tissue fixation system - sacrouterine ligament)
32	70	m	Bladder cancer G2 pT3b pN0 (0/15) MX V0 L0 R0 (squamous cell carcinoma)	Radical cystectomy/ileal conduit	S/P 1× TUR-bladder pT2 G3 (squamous cell carcinoma)
33	81	m	Bladder cancer ypTis pN0 (0/17) R0 cM0 Prostate cancer pT2a pN0 (0/17) R0 L0 V0, Gleason 3 + 3 = 6	Radical cystectomy/ileal conduit	S/P 1× TUR-bladder pT1 (m, is) G2-3
34	63	m	Cancer of prostatic duct	Radical cystectomy/ileal conduit	S/P mult. TUR-bladder (carcinoma in prostatic duct)
35	74	m	Bladder cancer pT1 pN0 cM0 R0 G2 V0 L0	Radical cystectomy/neobladder	S/P1× TUR-bladder at least pT1 G3
36	59	m	Bladder cancer G3 pT2(is) pN2 (3/25) pM1 (lymph) L1 V1 R0 Prostate adenocarcinoma G2, Gleason 3 + 3 = 6, pT2b pN0 L0 V0 Pn1 R0	Radical cystectomy/neobladder	S/P1× TUR-bladder mpT2 (is) L1 G2-3
37	76	m	Bladder cancer and prostate cancer pT2c pN0 (0/18) R0 L0 V0 pN0, Gleason: 3 + 3 = 6	Radical cystectomy/ileal conduit	no history available
38	68	w	Neurogenic bladder, multiple sclerosis	Cystectomy/ileal conduit	Chronic pelvic pain, detrusor hyperactivity, incomplete spastic paraplegia
39	51	m	Bladder cancer pT4a (is) pN0 (0/16) G3 R0 L1 V0 cM0	Radical cystectomy/ileal conduit	S/P 1× TUR-bladder G3, at least pT1 Nx M0 Nicotine abuse
40	84	m	Bladder cancer G3 pT3a (is) pN0 (0/11) L0 V1 R0 cM0 Prostate adenocarcinoma Gleason 4 + 4 = 8, G3 pT2c pN0 L0 V0 R0 cM0	Radical cystectomy/ileal conduit	no history available
41	74	m	Bladder cancer G3 pT1 pN0 (0/18) L0 V0 R0 M0	Radical cystectomy/ileal conduit	S/P G3, at least pT1 and carcinoma in situ.

*S/P* status post, *TUR* transurethral resection

### Quantitative RT-PCR analysis

Small pieces from the detrusor smooth muscle portion of approx. 1–2 mm width were prepared from the human tissue sample and immediately frozen in liquid nitrogen. Care was taken that these pieces for quantitative PCR were urothelium-free. For mRNA isolation, TRIZOL reagent was used, and total RNA was reverse-transcribed using Moloney murine leukemia virus reverse transcriptase (200 U/μL) and RNasin Plus RNase inhibitor (40 U/μL, both Promega Corporation, Madison, WI, USA) in the presence of random hexamers (3 μg/μL) and dNTP Mix (10 mmol/L each, Invitrogen, Carlsbad, CA, USA). For the real-time PCR of the target genes (Rho kinase 1 [ROCK1], Rho kinase 2 [ROCK2], myosin-light chain kinase [MLCK]) as well as three standard reference genes (glyceraldehyde-3-phosphate dehydrogenase [GAPDH], β-actin [ACTB], phosphoglycerate kinase 1 [PGK1]), we used the QuantiFast SYBR Green PCR Kit (concentration as recommended by the manufacturer, Qiagen Inc., Valencia, CA, USA). The mastermix was aliquoted, cDNA and primers (Cf = 20 μmol) were added. All primers purchased from Molbiol (Berlin, Germany) are given in Table 2. The reference genes ACTB and PGK1 were detected using Qiagen Primer Assays (Qiagen Inc., Valencia, CA, USA). The PCR product length was 156–285 bp (Table 2). Real-time PCR was performed using the ep mastercycler (software realplex 2.2, Eppendorf, Hamburg, Germany) with cycling parameters 95.0 °C for 2 minutes once, followed by 95.0 °C for 15 s and the annealing temperature for 15 s, with normalized fluorescence read at 68.0 °C (520 nm) for 40 cycles. The annealing temperatures were adjusted using gradient PCR (ROCK1/GAPDH 53.7 °C, ROCK1/ACTB/PGK1 57.5 °C, ROCK2/GAPDH 62.6 °C, ROCK2/ACTB/PGK1 57.5 °C). Single product amplification was confirmed by melting curve and gel electrophoresis analysis. Then, the efficiency was determined by PCR using serial dilution of the cDNA. Messenger-RNA (mRNA) expression levels were efficiency-corrected and determined by normalizing the target genes (ROCK1, ROCK2, MLCK) with three standard reference genes (GAPDH, ACTB, PGK1), expressed as the mean of 2^-∆∆Ct^ ± SEM.

**Table 2:** Forward and reverse primers of ROCK1, ROCK2, MLCK and GAPDH (from Molbiol). ACTB and PGK1 were detected with Qiagen Primer Assays

Gene name	Forward primer	Reverse primer	PCR product
ROCK1	AAAATTGTGTGAGGAGGACATGG	TTCATCCCAACATTCTTGGATCT	279 bp
ROCK2	GCAATGCGGTAAAAAGCGA	GGGAATCATGGTGTGACCAA	217 bp
MLCK	GTCTTATGTTATCTTCCATTCTA	TATAATAAACTGTGGCAATACTG	156 bp
GAPDH	AGAAGGCTGGGGCTCATTTG	AGGGGCCATCCACAGTCTTC	285 bp

### Statistics

All data are expressed as means ± SEM. Statistical comparison was performed using the two-tailed Student’s t-test (SigmaStat 3.5). The level of significance is indicated by asterisks (*P < 0.05; **P < 0.01).

## Results

**Fig. 1:**
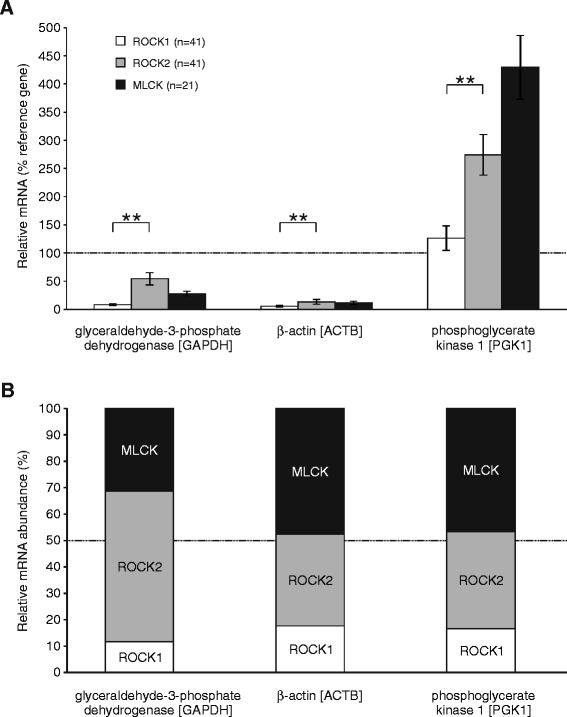
Expression levels of the contraction enzymes Rho kinase (isoforms ROCK1 and ROCK2) and myosin light-chain kinase (MLCK) in the human detrusor smooth muscle. **a** Relative mRNA content for target genes ROCK1 (white), ROCK2 (gray) and MLCK (black), expressed as percentage of the reference genes GAPDH, ACTB and PGK1. Note that ROCK2 was significantly more expressed than ROCK1. **b** Relative mRNA abundance of ROCK1, ROCK2 and MLCK. Note that expression level of ROCK1 and ROCK2 together was similar to that of MLCK

In the present study, we aimed to investigate the transcriptional expression of Rho kinase (ROCK, isoforms ROCK1 and ROCK2) and myosin-light chain kinase (MLCK) in human detrusor smooth muscle using quantitative RT-PCR, normalized to three commonly used reference genes (glyceraldehyde-3-phosphate dehydrogenase [GAPDH], β-actin [ACTB], phosphoglycerate kinase 1 [PGK1]). As it is shown in Fig. 1a, ROCK2 mRNA levels were significantly higher than ROCK1 mRNA levels (*P* < 0.01 for all three reference genes, paired two-tailed t-test) indicating that ROCK2 is the predominant Rho kinase isoform in human urinary bladder. The relative mRNA contribution was 15.2 ± 1.9 % for ROCK1, 42.8 ± 3.8 % for ROCK2 and 41.9 ± 4.8 % for MLCK (Fig. 1b). When pooling the relative mRNA quantification for ROCK1 and ROCK2, however, there was no consistent difference between ROCK (i.e. ROCK1 and ROCK2 together) and MLCK expression (*P* > 0.05 for ACTB and PGK1, *P* < 0.05 for GAPDH, unpaired two-tailed t-test) suggesting that ROCK and MLCK transcripts were almost equally abundant (Fig. 1b).

**Fig. 2:**
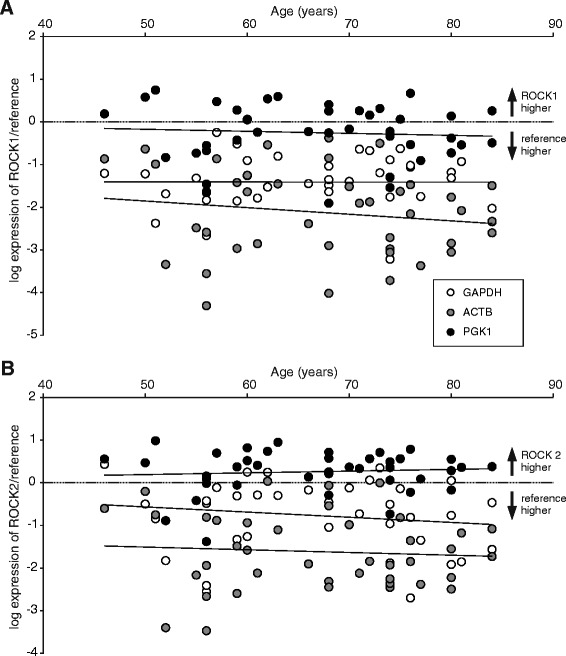
Expression of ROCK1 and ROCK2 is not correlated with age. Relative mRNA content for the target genes ROCK1 **(a)** and ROCK2 **(b)**, normalized to reference genes GAPDH, ACTB and PGK1 plotted against the patients’ age (*n* = 41). There was no significant correlation between any of these target genes and age

**Fig. 3:**
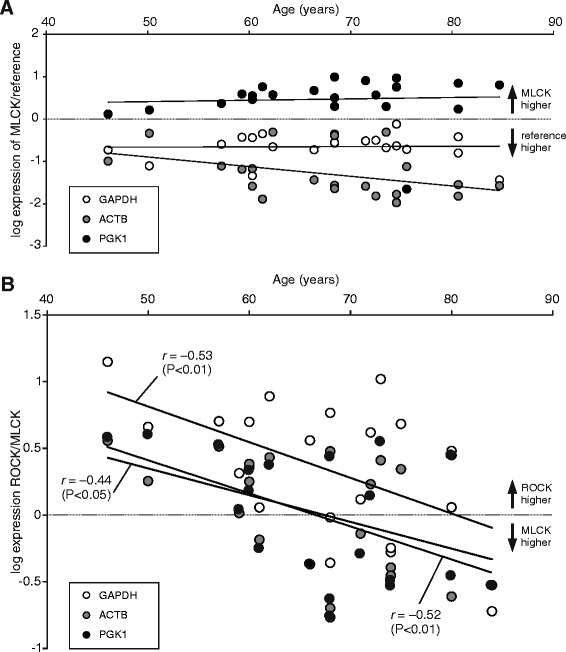
The ROCK-to-MLCK ratio is negatively correlated with age. **a** Relative mRNA content for the target gene MLCK, normalized to reference genes GAPDH, ACTB and PGK1 plotted against the patients’ age (*n* = 21). There was no consistent correlation between MLCK and age. **b** Ratio of ROCK expression to MLCK expression, plotted gainst age (*n* = 21). Note that there was a significant negative correlation between the ROCK-to-MLCK ratio and age using all three reference genes

Since we have previously found that Rho kinase (ROCK) contribution to cholinergic detrusor contraction increased with age [5], we hypothesized that expression of this enzyme might be up-regulated in the aging detrusor. In order to test this, we performed a linear correlation analysis between relative mRNA levels and age (Fig. 2a,b). However, over the broad range of ages from 46 to 84 years we could not detect a significant correlation between ROCK1 or ROCK2 to any reference gene used (Fig. 2a,b). The Pearson’s correlation coefficients for these bivariate analyses are given in Table 3. With respect to the other key enzyme of smooth muscle contraction, the correlation coefficient between MLCK normalized to ACTB and age reached statistical significance (Fig. 3a, Table 3), but it is obvious that MLCK expression levels did not consistently show age-dependent changes. One has to take into account that the vast majority of patients included in this study were cystectomized due to bladder cancer. Thus, mRNA expression of the analyzed contraction enzymes could be altered by the adjacent tumor infiltration. We therefore performed a regression analysis between the tumor size (pathological tumor size, pT1-4 in Table 1) and mRNA expression, but did not obtain significant correlation (data not shown). Since 33/41 (80 %) patients were male, we tested whether there was a gender specificity in our data. As also shown in Table 3, the correlation coefficients in this subgroup analysis of male patients were similar to those of the total study population. On the other hand, the subgroup analysis for female patients did not reveal statistically significant correlation coefficients either (data not shown).

**Table 3:** Pearson’s correlation coefficients between target gene (ROCK1, ROCK2 and MLCK, ROCK-to-MLCK ratios) and age using three different reference genes

Target gene	Reference genes		
	GAPDH	ACTB	PGK1
**ROCK1 (** ***n*** **= 41)**	−0.0043	−0.1584	−0.0732
only male subjects (*n* = 33)	−0.0375	−0.1577	−0.1596
**ROCK2 (** ***n*** **= 41)**	−0.1436	−0.0733	0.0812
only male subjects (*n* = 33)	−0.0884	−0.0404	0.0810
**MLCK (** ***n*** **= 21)**	0.0201	−0.4151*	0.0593
only male subjects (*n* = 18)	0.2899	−0.3549	0.0371
**ROCK1-to-MLCK ratio (** ***n*** **= 21)**	−0.2996	−0.3325	−0.3839*
only male subjects (*n* = 18)	−0.2802	−0.4389*	−0.5364*
**ROCK2-to-MLCK ratio (** ***n*** **= 21)**	−0.4757*	−0.4427*	−0.4298*
only male subjects (*n* = 18)	−0.4246*	−0.4694*	−0.5854**
**ROCK-to-MLCK ratio (** ***n*** **= 21)**	−0.5297**	−0.4352*	−0.5181**
only male subjects (*n* = 18)	−0.4625*	−0.4981*	−0.6126**

Correlation coefficients in bold indicate that statistical significance was reached using all reference genes (*P < 0.05, **P < 0.01; t-test)

So far, we have not been able to find robust age-dependent differences in mRNA abundance for ROCK1, ROCK2 or MLCK. In these interindividual analyses, however, we have plotted each individual against age and may have missed differential changes of these enzymes within a given subject. We, therefore, calculated the ROCK-to-MLCK ratio of every subject and plotted this ratio against age. As shown in Fig. 3b, this analysis revealed a decrease of the ROCK-to-MLCK ratio during aging. This negative correlation was significant using all three reference genes (Fig. 3b). Thus, we conclude that there is a robust age-dependent drop of the ROCK-to-MLCK ratio in the aging human detrusor smooth muscle. Since ROCK2 was the predominant isoform in human detrusor, we also calculated the correlation coefficients for both isoforms separately (Table 3). These analyses revealed that ROCK2-to-MLCK ratio is consistently inversely related to age, but not the ROCK1-to-MLCK ratio (Table 3). These data also suggest that ROCK2 and MLCK are generally negatively correlated. On average, the correlation coefficient between ROCK2 and MLCK mRNA levels was *r* = −0.5541 (*n* = 21, *P* < 0.01, two-tailed t-test). In summary, across all subjects we found an inverse relationship between ROCK and MLCK transcription, respectively. Within a given subject, however, aging was associated with a relative ROCK down-regulation and concomitant MLCK up-regulation.

## Discussion

Smooth muscle contraction involves activation of two key enzymes – Rho kinase (ROCK) and myosin light-chain kinase (MLCK). Both have been demonstrated in human detrusor contraction [5], and the present study was performed in order to quantify the mRNA levels for the both isoforms of Rho kinase (ROCK1 and ROCK2) as well as for MLCK in the aging human detrusor smooth muscle.

Quantified PCR data do necessarily depend on the choice of reference genes used for normalization. In particular, altered expression of a reference gene is a potential pitfall in PCR studies that may substantially contaminate the quantification of the genes of interest. With respect to the present study, selection of reference genes in human bladder tissue primarily derived from bladder cancer patients is not simple [6]. Although we have used macroscopically tumor-free tisse specimens, reference genes could be altered by the adjacent pathology. First, we have used the most commonly used reference gene, glyceraldehyde-3-phosphate dehydrogenase (GAPDH). This, however, may be up-regulated in bladder cancer [7]. Hence, we also used β-actin (ACTB) which was not significantly altered in bladder cancer [6] or prostate cancer [8]. The last reference gene, the glycolysis enzyme phosphoglycerate kinase 1 (PGK1), is a target gene of both the myc oncogene pathway and the hypoxia inducible factor 1α (HIF-1α) and is therefore considered as a marker gene for a number of malignant tissues such as kidney cancer [9] or colon cancer [10]. Recently, HIF-1α activation and subsequent PGK1 up-regulation has also been demonstrated in bladder cancer [11]. Taken together, cancer-associated up-regulation of the reference genes used in the present study was (i) definitely present (PGK1), (ii) vaguely present (GAPDH) or (ii) absent (ACTB). As a consequence, altered target gene expression was only accepted to be relevant when it could consistently be observed with all three reference genes.

Obviously, the target genes may also be altered by the malignant pathology present in the vast majority of cases. While care was taken to dissect tissue from the macroscopically healthy bladder wall with non-infiltrated urothelium, we cannot exclude the enzyme expression to be altered in detrusor tissue from bladder cancer patients. At least, the non-selective ROCK inhibitor HA-1077 has been found to be beneficial against urothelial cancer [12] and this finding might be interpreted as an indication for an enhanced expression of this enzyme in bladder cancer. However, we did not observe a significant correlation between tumor size and ROCK1 or ROCK2 mRNA expression.

First, our data demonstrated that transcript levels for ROCK (i.e. ROCK1 and ROCK2 together) were similar to that for MLCK. Hence on the transcriptional level, both key enzymes were equally expressed. This is important to note, since it is consistent with functional data from cholinergic contractions of human detrusor specimens that showed an equal pharmacological effect of ROCK and MLCK inhibition, respectively [5]. Thus, both enzymes are not only equally transcribed, but also equally involved on the functional level. Moreover, the present study has also shown that ROCK2 mRNA is significantly more abundant than ROCK1 mRNA. Hence, the predominant Rho kinase isoform in the human detrusor is ROCK2. This is in contrast to rat detrusor tissue, which showed equal transcript levels for ROCK1 and ROCK2 [13]. The predominance of ROCK2 in the human detrusor is a clinically relevant finding, since Rho kinase inhibitors are currently developed for different potential indications, and research so far has largely been concentrated on the ROCK1 isoform – in particular in vascular smooth muscle. The notion that this isoform is implicated in arterial hypertension [14] has launched substantial pharmaceutical interest in developing ROCK1 inhibitors as a new class of antihypertensive medication [15]. Moreover, increased ROCK function was also found to be involved in pulmonary hypertension, and again transcription levels of ROCK1 were more markedly enhanced than transcription levels of ROCK2 in both rat [16] and human [17]. Currently, specific ROCK2 inhibitors are not available, but the present study suggests that ROCK2 might be regarded as potential therapeutic target in overactive bladder syndrome.

Another major finding was that the ROCK-to-MLCK ratio showed a significant negative correlation with aging, even though there was no correlation between any of these genes and age. In other words, within a given subject, there was a relative ROCK down-regulation and – at the same time – MLCK up-regulation. This was clearly an unexpected finding, since ROCK contribution to contraction increased with aging as opposed to the contribution of MLCK [5]. Given the fact that the down-regulation of ROCK in the aged detrusor was primarily due to down-regulation of ROCK2, one can speculate about the mechanism of this age-dependent transcriptional alteration. We already know from rodent studies that Rho kinase is involved in bladder hyperactivity [18], but in addition, it was always the ROCK1 isoform that was identified to be up-regulated in rat decompensated detrusor hypertrophy [19] or in altered detrusor contractility of diabetic rabbits [20]. Thus, down-regulation of ROCK2 in aged human detrusor tissue is therefore a rather uncommon example of transcription regulation, and possibly not the primary cause. It is therefore intriguing to speculate whether transcriptional ROCK2 down-regulation might be secondary in order to compensate for an enhanced Rho kinase function in the aging bladder. Further studies are needed to address this question experimentally.

## Conclusion

The present study shows that ROCK and MLCK are inversely regulated and that down-regulation of ROCK – and in particular ROCK2 – as occurs during aging is counterbalanced by an up-regulation of MLCK. In conclusion, we suggest that there is a physiological homeostatic regulation of both enzymes which is probably disturbed in the aged detrusor. Therefore, pharmacological intervention such as specific ROCK2 inhibitors might be beneficial to reduce bladder overactivity in the elderly.

## Abbreviations

ACTB: β-actin
GAPDH: Glyceraldehyde-3-phosphate dehydrogenase
MLCK: Myosin-light chain kinase
mRNA: Messenger-RNA
PGK1: Phosphoglycerate kinase 1
ROCK: Rho kinase
RT-PCR: Real-time reverse transcriptase polymerase chain reaction
